# Cortisol-to-DHEAS awakening response ratio in people with dementia and family caregivers: Associations with age, dementia severity and agitation

**DOI:** 10.1016/j.cpnec.2025.100334

**Published:** 2025-12-19

**Authors:** Wanrui Wei, Töres Theorell, Gabriella Engstrom, Azita Emami

**Affiliations:** a School of Nursing, Yale University, 400 West Campus Drive, Orange, CT, 06477, United States; b Division of Global Health, Karolinska Institutet, Stockholm, Sweden; c Stress Research Institute, Department of Psychology, Stockholm University, Stockholm, Sweden; d School of Health and Welfare, Dalarna University, SE-791 88, Falun, Sweden; e Division of Occupational Therapy, Department of Neurobiology, Care Sciences, and Society, Karolinska Institutet, Fack 23200, 141 83, Huddinge, Sweden

**Keywords:** Agitation, Awakening response, Cortisol, Dementia, Dehydroepiandrosterone, Saliva

## Abstract

**Background:**

The ratio of cortisol to dehydroepiandrosterone sulfate (DHEAS) in the awakening response has emerged as a potential biomarker of stress-related dysregulation in neurodegenerative conditions. Whether this ratio differs between people with dementia (PWD) and family caregivers, and how it varies with age, sex, dementia severity and agitation, remains unclear.

**Methods:**

We analyzed 1093 day-level saliva samples from 58 participants (PWD = 28; caregivers = 30). The primary outcome was the log-transformed awakening response ratio of cortisol to DHEAS. Linear mixed-effects models with a participant random intercept and natural splines for age estimated group contrasts as geometric mean ratios (GMRs) from estimated marginal means. Fixed-effect predictors included age, sex, dementia severity (Global Deterioration Scale, GDS), and agitation (Brief Agitation Rating Scale, BARS). Model comparisons were conducted. Within PWD, mean-centered models tested one interaction at a time (Sex × GDS, Sex × BARS, Age × GDS, Age × BARS).

**Results:**

There was no between-group difference in the ratio after accounting for within-participant clustering and age (GMR = 0.97, 95 % CI 0.64–1.46; *p* = 0.87). Within PWD, interaction models indicated that the association between age and the ratio strengthened with higher dementia severity (*β* = 0.043, *p* = 0.04) and greater agitation (*β* = 0.011, *p* = 0.006). Marginal R^2^ ranged 0.114–0.141; conditional R^2^ 0.358–0.376.

**Conclusions:**

Although average ratio did not differ between PWD and caregivers, it increased more steeply with age at higher dementia severity and agitation. These findings highlight the cortisol-to-DHEA(S) awakening response ratio as a non-invasive and clinically relevant biomarker reflecting symptom-linked neuroendocrine heterogeneity in dementia.

## Highlights

• This study is the first to examine the cortisol-to-DHEAS awakening response ratio in people with dementia.
• No average difference in the ratio between people with dementia and family caregivers.
• Age–ratio association strengthens at higher dementia severity.
• Age is more positively related to the ratio when agitation is higher.

## 1 Introduction

Dementia ranks as the seventh leading cause of mortality worldwide and is a primary contributor to disability and dependency among older people, with nearly 10 million new cases diagnosed annually [1]. An estimated 9.8 million individuals in total European countries live with dementia, a number expected to reach more than 18.8 million by 2050 [2]. Although the precise neuropathological pathways underlying Alzheimer's disease (AD) remain under investigation, increasing evidence points to chronic stress as a key modifiable risk factor [3]. Individuals reporting elevated levels of perceived stress have shown a significantly increased likelihood of developing AD [4]. In community-based settings, both people living with dementia (PWD) and their family caregivers are frequently exposed to high and sustained levels of psychological stress [5]. Compared to non-dementia caregivers or non-caregivers, dementia caregivers are consistently found to experience greater emotional distress, depressive symptoms, and caregiving burden [[6], [7], [8], [9]].

Cortisol and dehydroepiandrosterone (DHEA) and its sulfate (DHEAS) are commonly assessed as indicators of physiological stress [10]. Stress exposure activates the hypothalamic–pituitary–adrenal (HPA) axis, with corticotropin-releasing hormone (CRH) stimulating pituitary adrenocorticotropic hormone (ACTH), which in turn drives adrenal secretion of both cortisol and DHEA(S) [[11], [12], [13]]. Because both steroids are ACTH-dependent, changes in one relative to the other provide a readout of adrenocortical secretory regulation, wherein one hormone may be up- or down-regulated over time in response to chronic stress [10]. Despite being co-synthesized, released, and converted by a similar set of prohormones in response to stress, cortisol and DHEA(S) exert contrasting effects across neurological, immune, and metabolic systems [11]. Consequently, rather than focusing solely on their absolute concentrations, the cortisol-to-DHEAS ratio has been proposed as a more informative and relative measure, capturing the balance between catabolic and anabolic stress responses [14]. This ratio is increasingly recognized as a reflection of HPA axis homeostasis, with implications for aging, neurodegeneration, and resilience to stress-related pathology [3].

Cortisol typically rises robustly within the first hour after awakening—a phenomenon known as the cortisol awakening response, which is considered a reliable marker of HPA axis functioning [15]. The cortisol awakening response is associated with the HPA axis and brain functions of the same day in healthy subjects [10]. While DHEA does not exhibit a parallel awakening surge, its early morning levels are consistently higher than those later in the day [16]. Therefore, the cortisol-to-DHEAS awakening response ratio may provide a stable, integrative index of endocrine stress regulation. To our knowledge, prior work has examined the cortisol awakening response and used non-awakening cortisol-to-DHEA(S) ratios; however, the awakening response of the ratio—particularly in dementia populations—remains underexplored.

Age-related changes in endocrine regulation are well documented, including elevated basal cortisol levels, reduced sensitivity to negative feedback, and diminished secretion of DHEA(S) and aldosterone [17]. Hormonal aging is increasingly recognized as a dynamic and potentially nonlinear process, encompassing age-related changes in HPA-axis tone (elevated basal cortisol, blunted negative feedback) and curvilinear declines in DHEA(S) with advancing age [18,19]. These shifts highlight the importance of accounting for age when examining stress-related hormonal biomarkers. Hence, it is critical to consider age when investigating cortisol and DHEAS levels. Sex differences are relevant to both dementia prevalence and DHEAS secretion [20,21], and males generally show higher circulating DHEAS levels than females across the lifespan [22]. While animal models often report heightened cortisol responses in females following HPA-axis activation, human studies have yielded inconsistent findings [17]. Given these trajectories, the cortisol-to-DHEAS awakening response ratio is likely to vary non-monotonically with age and may differ by sex. We therefore stratify by age and model non-linear effects to obtain unbiased estimates. This study addresses that gap by investigating group differences and non-linear hormonal trajectories across demographic subgroups.

A persistent alteration of the HPA axis has been documented in AD [23]. In this axis, CRH from the hypothalamus stimulates pituitary ACTH, which then drives adrenal glucocorticoid secretion [23]. Experimental work indicates that HPA-axis activation can precede overt symptoms. Young 3xTg-AD mice exhibit an activated HPA axis at early neuropathological stages, despite normal basal glucocorticoid levels, and periodic cognitive stimulation elevates corticosterone, accompanied by greater amyloid-β pathology, suggesting a stress-responsive loop that sustains axis dysregulation [24]. Elevated baseline plasma cortisol predicts accelerated hippocampal atrophy, and reduced hippocampal volume in turn confers greater risk of conversion to AD, consistent with an HPA-neurodegeneration pathway at prodromal stages [25].

During AD progression, HPA-axis dysregulation and the resulting excess cortisol may facilitate neuronal dysfunction and synaptic depletion. Cumulatively, this sequence is considered to worsen behavioral disturbances and cognitive deterioration in individuals with AD [23]. Furthermore, agitation is a behavioral syndrome characterized by increased motor activity, irritability, and emotional distress, with multifactorial etiologies that include medical, psychiatric, and environmental stressors [26]. Taken together, these observations indicate stress-endocrine dysregulation across the AD course. Accordingly, we prespecified dementia severity and agitation as clinically informative correlates and examined their associations with the awakening response of the cortisol-to-DHEAS ratio.

To our knowledge, there has been no empirical study of the awakening responses of cortisol and DHEAS ratio in PWD. This study aims to: (i) assess whether the cortisol-to-DHEAS awakening response ratio differs between PWD and family caregivers without dementia; (ii) test whether age and sex modify the PWD–caregiver contrast in awakening response of the cortisol-to-DHEAS ratio; and (iii) examine associations of dementia severity and agitation with the awakening response of the cortisol-to-DHEAS ratio among PWD. Family caregivers served as cognitively healthy, age- and household-matched comparators who also face sustained stress, providing a high-stress reference to test whether disease-specific HPA dysregulation in PWD persists beyond shared stress exposure. Our hypothesis was that, given comparable levels of chronic stress, greater HPA-axis dysfunction—indexed by a higher awakening response of the cortisol-to-DHEAS ratio—would be associated with poorer clinical profiles. Accordingly, we expected that PWD would exhibit greater evidence of HPA axis dysregulation, as reflected in altered awakening response ratios, compared to family caregivers. We also anticipated that agitation and dementia severity would be positively associated with the cortisol-to-DHEAS awakening response ratio in PWD.

## 2 Methods

### 2.1 Participants & procedure

This secondary analysis utilized data from an intervention study [27,28], drawing on all available awakening saliva samples from the parent intervention trial. As demonstrated in a recent methodological evaluation, the sample size was deemed sufficient to detect a treatment effect exceeding 5 % [29]. Participants were recruited by telephone through a memory evaluation center serving a socioeconomically diverse community. Eligible individuals were community-dwelling PWD who had a spouse or family member acting as their primary caregiver. Screening for eligibility was conducted during a face-to-face meeting with an occupational therapist. Inclusion criteria for PWD: 1) A clinical diagnosis of dementia established by a physician; 2) Living at home with a family caregiver able to assist with or independently complete saliva collection; 3) A Global Deterioration Scale (GDS) rating between 4 and 7 [30], indicating moderate to severe dementia; 4) A Brief Agitation Rating Scale (BARS) score ≥15 [31]. Exclusion criteria for PWD: 1) Diagnosis of frontotemporal dementia or traumatic brain injury, due to potential impacts on emotional processing; 2) Active, untreated pain, infection, or other medical conditions known to contribute to neuropsychiatric symptoms. For the present analysis, one family caregiver participant (a 37-year-old male) was excluded due to age-based outlier status.

Saliva samples were collected at home by the participants for eight consecutive weeks following validated home-based protocols to ensure feasibility and consistency. Each participant was instructed to collect two saliva samples per morning—immediately upon awakening (Sample 1) and 15 min post-awakening (Sample 2)—across five weekdays from Sunday evening to Friday morning. Although weekend samples were not explicitly required, a small number were collected and included in the analysis. Participants followed their natural waking routines, and collection was not restricted to specific clock times. Awakening saliva Sample 2 was collected at 15 min to reduce burden and maximize adherence. This early window was prespecified to index initial HPA-axis mobilization, acknowledging that the later 30–45-min peak of the cortisol awakening response was not captured.

Data collection occurred from November 2018 to March 2020. The study received ethical approval from the Karolinska Institutet Institutional Review Board, Stockholm, Sweden (Dnr: 2018/1596-31/2). Written informed consent was obtained from family caregivers, and assent or proxy consent from the caregiver was obtained for PWD.

### 2.2 Measures

#### 2.2.1 Demographics and health characteristics

Demographic information included age, sex, and participant role (PWD vs. family caregiver).

Dementia severity was assessed using GDS [30], a widely used seven-stage clinical tool for rating the progression of cognitive decline. Stages 1–3 represent pre-dementia phases, including subjective forgetfulness and mild cognitive impairment, whereas Stages 4–7 correspond to dementia with increasing loss of functional independence. Beginning in Stage 5, individuals typically require daily assistance. Each stage includes a numeric score, a descriptive label, and structured behavioral criteria. For the present analyses, dementia severity was categorized as mild dementia (GDS = 4), moderate dementia (GDS = 5), and moderately severe/severe dementia (GDS = 6–7). The GDS has been validated in both clinical and research settings and is widely used to characterize dementia severity and disease progression.

Neuropsychiatric symptoms (agitation) were evaluated using BARS [31], a 10-item measure adapted from the Cohen-Mansfield Agitation Inventory. Items are rated on a 7-point frequency scale ranging from 1 (none) to 7 (several times per day), reflecting behaviors observed in the past two weeks, yielding a total score between 10 and 70. Higher scores indicate more frequent and severe agitation. BARS has demonstrated good psychometric properties in nursing home populations with dementia, including internal consistency (Cronbach's α = 0.74–0.82), inter-rater reliability (intraclass correlation coefficient [ICC] = 0.73), and significant concurrent validity with instruments such as the Behavioral Pathology in Alzheimer's Disease and the Behavioral Syndromes Scale for Dementia.

These variables were treated as predictors in hormonal response models.

#### 2.2.2 Saliva hormones

Salivary cortisol and DHEAS levels were assayed from morning saliva samples. Two validated collection methods were employed: the SalivaBio Children's Swab (SCS) for PWD and the passive drool method using a Saliva Collection Aid (SCA) for family caregivers. Both were obtained from Salimetrics (State College, PA) [32,33]. Prior work shows no systematic concentration differences across Salivette, passive drool, and SalivaBio under home-sampling conditions, supporting cross-method comparability [34]; furthermore, swab-based saliva can predict serum total/free cortisol as well as or better than passive drool [35]. For individuals with cognitive impairment, the SCS facilitated compliance by allowing participants to gently chew the swab for 1 min before transferring it to a storage tube [36,37]. Apart from this, no efforts were made to stimulate salivation. Family caregivers used the passive drool method, which is considered the gold standard, by passively collecting saliva into a cryovial.

All collection tubes were pre-labeled with barcodes indicating participant ID and sample timing (e.g., first-morning tube for Monday). Study coordinators provided detailed orientation for each dyad, including study purpose, collection protocol, and sample pick-up procedures. During the collection period, trained research assistants delivered collection kits, retrieved samples daily, and verified labeling accuracy. Participants were instructed to place collected samples in sealed plastic bags and refrigerate them immediately. Assistants transported samples in temperature-controlled refrigerated biological containers to the Karolinska Institutet Biobank, where they were centrifuged and stored until shipment for laboratory analysis.

To enhance adherence—defined as completing the instructed five weekday saliva sample collections within every week—the study coordinator made weekly reminder calls. Participants could report collection issues, and home visits were offered as needed. Missing or invalid samples were documented and excluded from final analyses. This collection protocol has been previously validated in studies involving community-dwelling older adults with dementia [5,28,38]. The final analytic sample excluded participants with incomplete pairs or missing values. The following derived indices were computed:

Awakening Response Cortisol (ARC)*: Cortisol Sample 2/Cortisol Sample 1*.

Awakening Response DHEAS (ARDHEAS)*: DHEAS Sample 2/DHEAS Sample 1*.

Awakening Response Cortisol to Awakening Response DHEAS Ratio*: ARC/ARDHEAS*.

### 2.3 Statistical analysis

All statistical analyses were performed using R version 4.2.3. Observations with missing data were deleted. Log-transformations were applied to biomarker variables before analysis to improve distributional properties and interpretability [39,40]. Normality was assessed via Shapiro–Wilk tests and Q–Q plots, and homogeneity of variance was examined using Levene's test. The primary outcome variable was computed as the log-transformed ratio of awakening response cortisol to awakening response DHEAS (log[ARC/ARDHEAS]). Because awakening responses were collected on multiple days per participant, day-level associations between log(ARC/ARDHEAS) and group, demographic variables, and, in PWD-only models, clinical characteristics were examined using linear mixed-effects models (LMMs) with random intercepts for participant ID to account for within-person clustering. Group contrasts and covariate effects were obtained from the fitted models on the log scale and reported on the original scale as geometric mean ratios (GMRs) with 95 % confidence intervals (CIs) after back-transformation. Detailed model specifications, contrast procedures, stratified analyses, robustness checks, and PWD-only clinical predictor models are described in the following sections.

#### 2.3.1 Mixed-effects model specification

We fitted LMMs with a random intercept for participant ID to account for intra-individual clustering across days. The main analytic model was:

log(ARC/ARDHEAS)=Group+Sex+ns(Age,df=4)+(1|ID)

Fixed effects were Group (PWD vs. family caregiver), Sex, and Age modeled as a natural spline (ns) with four degrees of freedom (df) to flexibly capture non-linear age trends. The model was estimated using restricted maximum likelihood (REML) for reporting coefficients, while maximum likelihood (ML) estimation was used for model comparisons with Akaike's Information Criterion (AIC) and likelihood-ratio tests (LRTs). Model performance was summarized by Nakagawa's marginal R^2^ (variance explained by fixed effects) and conditional R^2^ (variance explained by fixed and random effects combined), with ICCs derived from the random-effects variance components.

#### 2.3.2 Contrasts and effect estimation

The primary contrast of interest was between-group difference (PWD vs. caregiver) in log(ARC/ARDHEAS). Estimated marginal means were obtained from the fitted LMMs on the log scale and compared using pairwise contrasts. Contrasts were back-transformed to the original ratio scale and reported as GMRs with 95 % CIs and two-sided *p* values. A GMR = 1 indicates no group difference; GMR<1 indicates a lower mean in PWD; and GMR>1 indicates a higher mean in PWD. Model comparison between linear and spline representations of age used ML-based AIC and LRT.

#### 2.3.3 Stratified and robustness analyses

We presented sex-stratified and age-stratified contrasts (median split at 76 years at the participant level) while retaining continuous ns (Age, df = 4) in the model.

Robustness of the main GMR estimate was examined using a series of sensitivity analyses. These included: (i) removal of single-day IDs, (ii) leave-one-participant-out (LOCO) re-estimation, (iii) trimming 1 % two-sided tails in log outcome, (iv) removing day-level observations with |Pearson residual|>3, (v) removing the two most influential participants per LOCO *p*-value ranking, (vi) specification checks replacing ns(Age, df = 4) with linear or alternative spline bases (df = 3 or 5), and (vii) parametric bootstrap (199 runs) for the GMR.

#### 2.3.4 PWD-only models of clinical predictors

We analyzed repeated awakening-response days nested within PWD. To aid interpretation of interaction terms and reduce collinearity, continuous predictors were mean-centered within the PWD subset (Age_c_, GDS_c_, and BARS_c_). The base model included Sex, Age_c_, GDS_c_, and BARS_c_ as fixed effects and a random intercept for ID:

$$\log\left(\text{ARC}/\text{ARDHEAS}\right)=\text{Sex}+{\text{Age}}_{c}+{\text{GDS}}_{c}+{\text{BARS}}_{c}+\left(1|\text{ID}\right)$$

We then introduced plausible interaction terms (Sex × GDS, Sex × BARS, Age × GDS, and Age × BARS) individually and compared each extended model to the base model using ML-based AIC and LRTs. Interactions that significantly improved fit were retained for final inference.

Final models were refitted using REML with Kenward–Roger small-sample corrections for Type-III tests. Fixed-effect estimates, associated standard errors, and *p* values are reported for retained interactions. Model performance was summarized with Nakagawa's R^2^ (marginal and conditional) and the ICC from a random-intercept-only model.

All models were implemented in *lme4* and *lmerTest*. Post-hoc contrasts used *emmeans*, and R^2^ statistics used the *performance* package. All tests were two-tailed, with a significance level set at *α* = 0.05.

## 3 Results

### 3.1 Participant characteristics

Fifty-eight participants (PWD: *n* = 28; family caregivers: *n* = 30) contributed 1093 day-level valid saliva samples and completed baseline assessments; five additional records were excluded during preprocessing. Mean age was 77.75 years (SD = 7.99) among PWD and 75.93 years (SD = 8.63) among caregivers (*p* = 0.41). The proportion of females was higher among caregivers (63.3 %) than among PWD (39.3 %), although this difference did not reach statistical significance (*p* = 0.058). Intervention allocation was balanced across groups, with no between-group difference in assignment proportions (*p* = 1.000). Full descriptive statistics are reported in Table 1.

**Table 1:** Participant characteristics.

		PWD (*n* = 28)	Family caregivers (*n* = 30)	*p* value
Age, years (mean (SD))		77.75 (7.99)	75.93 (8.63)	0.41
Intervention group, *n* (%)	Intervention	16 (57.1 %)	18 (60.0 %)	1.00
	Control	12 (42.9 %)	12 (40.0 %)	
Sex, *n* (%)	Female	11 (39.3 %)	19 (63.3 %)	0.06
	Male	17 (60.7 %)	11 (36.7 %)	
Dementia Severity, *n* (%)^a^	Mild dementia	15 (55.6 %)	–	–
	Moderate dementia	6 (22.2 %)	–	–
	Moderately severe or severe dementia	6 (22.2 %)	–	–
Agitation score, mean (SD)		26.11 (5.26)	–	–

*Note.* Continuous variables are summarized as mean (SD); categorical variables as counts (percent).

a GDS data were available for 27 of 28 PWD participants (one missing value).

We next compared groups on the log-transformed ratio of the awakening response cortisol to awakening response DHEAS, log(ARC/ARDHEAS). Group medians were 0.57 for PWD and 0.52 for caregivers (*p* = 0.61). For completeness, raw components (ARC and ARDHEAS) are also shown. These summaries are presented in Table 2.

**Table 2:** Between-group comparisons for log(ARC/ARDHEAS) and component measures.

Variable	*n* (PWD)	*n* (Caregiver)	PWD (Mean ± SD/Median [IQR])	Caregiver (Mean ± SD/Median [IQR])	*p* value
log(ARC/ARDHEAS)	505	588	0.57 [-0.24, 1.24]	0.52 [-0.34, 1.32]	0.61
ARC	505	588	0.95 [0.60, 1.76]	0.88 [0.51, 1.36]	<0.001
ARDHEAS	505	588	0.61 [0.31, 1.15]	0.52 [0.28, 0.96]	0.03

*Note.* Values are presented as mean ± SD for normally distributed variables and median [IQR] for non-normally distributed variables. *n* refers to the number of samples.

### 3.2 Group comparison of awakening hormone ratios (ARC/ARDHEAS)

#### 3.2.1 Primary mixed-effects analysis

Using linear mixed-effects models with a random intercept for participant and natural splines for age (df = 4) as a covariate, we found no evidence of a difference in log(ARC/ARDHEAS) between PWD and family caregivers after accounting for clustering and covariates (overall geometric mean ratio, GMR = 0.97; 95 % CI 0.64–1.46; *p* = 0.87). The intraclass correlation coefficient (ICC≈0.26) indicated substantial within-participant dependence, supporting the use of mixed-effects modeling.

Although a spline was retained to allow for potential non-linearity, the spline formulation did not outperform a linear age term (AIC 3453.1 vs. 3453.0; LRT *p* = 0.12). Model fit indices suggested modest fixed-effect explanatory power alongside meaningful clustering (Nakagawa's R^2^: marginal = 0.045, conditional = 0.282; ICC = 0.248).

#### 3.2.2 Stratified contrasts by age and sex

For context, the age-band distribution was well covered: at the day (sample) level, there were 351 caregiver and 228 PWD observations for participants <76 years, and 237 caregiver and 277 PWD observations for participants ≥76 years; at the participant level, the corresponding counts were 15 family caregivers and 12 PWD <76 years, and 15 caregivers and 16 PWD ≥76 years. Estimated marginal means from the primary model showed overlapping age profiles for PWD and caregivers across the adult lifespan, consistent with the non-significant contrast (see Supplementary Fig. 1 and Table 3).

**Table 3:** PWD vs caregiver contrasts (overall and stratified by sex and age) from LMM.

Outcome	Stratum	GMR	95 % CI	*p* value
log(ARC/ARDHEAS)	Overall (ns(Age,df = 4))	0.97	[0.64, 1.46]	0.87
log(ARC/ARDHEAS)	Sex: Female (ns(Age,df = 4))	0.81	[0.45, 1.47]	0.49
log(ARC/ARDHEAS)	Sex: Male (ns(Age,df = 4))	1.15	[0.63, 2.09]	0.64
log(ARC/ARDHEAS)	Age: <76 (ns(Age,df = 4))	1.12	[0.61, 2.05]	0.70
log(ARC/ARDHEAS)	Age: ≥76 (ns(Age,df = 4))	0.83	[0.48, 1.44]	0.50

We next examined whether the overall null finding masked differences by sex or age (see Table 3). Sex-specific contrasts remained non-significant, with a GMR of 0.81 (95 % CI 0.45–1.47; *p* = 0.49) for females and 1.15 (95 % CI 0.63–2.09; *p* = 0.64) for males, and the Group × Sex interaction did not improve fit (LRT *p* = 0.38). Age-specific contrasts defined by a median split at 76 years were likewise non-significant (<76 years: GMR = 1.12, 95 % CI 0.61–2.05, *p* = 0.70; ≥76 years: GMR = 0.83, 95 % CI 0.48–1.44, *p* = 0.50), and the Group × Age-category interaction was not supported (LRT *p* = 0.25).

#### 3.2.3 Sensitivity checks

Robustness analyses consistently reproduced the null group effect. LOCO analyses yielded overall GMRs between 0.92 and 1.03 with *p* values from 0.667 to 0.985, indicating that no single participant drove the findings. Parametric bootstrap resampling (199 draws) produced a mean GMR of 0.98 with a 95 % CI of 0.82–1.16. Additional checks—excluding single-day participants, trimming the 1 % tails, removing observations with |Pearson residual| > 3, dropping two influential IDs, and re-fitting models with linear age or alternative spline degrees (df = 3/5)—all returned GMRs close to 1 with confidence intervals spanning unity. Full results of these sensitivity and specification checks are provided in Supplementary Table 1.

### 3.3 Clinical predictors of ratio among people with dementia

Analyses were then restricted to PWD only (27 unique IDs; 478 person-days) to evaluate clinical predictors of day-level variability in log(ARC/ARDHEAS). The base random-intercept model included Sex, centered Age (Age_c), centered dementia severity (GDS_c), and centered agitation (BARS_c) as fixed effects. Candidate two-way interactions were screened one at a time using maximum-likelihood AIC and LRTs, followed by final estimation with REML and Kenward–Roger Type-III tests.

Screening showed that adding Age × GDS improved fit relative to the base model (AIC 1483.8 vs. 1487.6; LRT *p* = 0.02), and adding Age × BARS improved fit further (AIC 1479.6 vs. 1487.6; LRT *p* = 0.002). In contrast, neither Sex × GDS nor Sex × BARS improved fit (AIC 1487.6, *p* = 0.19; and AIC 1490.0, *p* = 0.76, respectively). The AIC and LRT screening summary appears in Table 4.

**Table 4:** Screening of candidate interaction terms in PWD (AIC and likelihood-ratio tests vs. Base).

Model	AIC	LRT *p* vs Base
Base	1487.6	–
Base + Sex × GDS	1487.6	0.19
Base + Sex × BARS	1490.0	0.76
Base + Age × GDS	1483.8	0.02
Base + Age × BARS	1479.6	0.002

Notes: We fit linear mixed-effects models (random intercept for participant ID) with the following base fixed-effects structure: logRR ∼ Sex + Age_c_ + GDS_c_ + BARS_c_ + (1|ID); ML where Age, GDS, and BARS were centered (subscript c).

In the model retaining Age × GDS, the interaction coefficient was positive and statistically significant (*β* = 0.043 per 1-year × 1-GDS-unit, SE = 0.019, *p* = 0.04). Type-III tests corroborated the interaction (F = 4.97, *p* = 0.04), while the main effects of age and GDS were not significant at their centered means. These estimates indicate that the association between older age and higher log(ARC/ARDHEAS) becomes increasingly positive as dementia severity increases; at average GDS the age slope is small and non-significant, but it steepens at higher GDS levels (see Table 5 for coefficients and Supplementary Table 2 for Type-III tests).

**Table 5:** Final REML estimates for PWD models with a single interaction (Age × GDS).

Fixed effect	*β* (Estimate)	SE	df	t	*p*
Intercept	0.611	0.251	24.09	2.44	0.02
Sex (Male)	−0.018	0.318	22.96	−0.06	0.96
Age_c	0.016	0.020	18.99	0.83	0.42
GDS_c	0.150	0.213	19.93	0.71	0.49
BARS_c	−0.040	0.037	21.85	−1.08	0.29
Age_c × GDS_c	0.043	0.019	17.59	2.23	0.04

In the model retaining Age × BARS, the interaction was likewise positive and statistically significant (*β* = 0.011 per 1-year × 1-BARS-point, SE = 0.004, *p* = 0.006), with confirmation by Type-III testing (F = 9.46, *p* = 0.007). This pattern suggests that the relation between age and log(ARC/ARDHEAS) strengthens with higher agitation; at the mean BARS value the age slope is small and non-significant, but becomes more positive as BARS increases (coefficients in Table 6, Type-III tests in Supplementary Table 3).

**Table 6:** Final REML estimates for PWD models with a single interaction (Age × BARS).

Fixed effect	*β* (Estimate)	SE	df	t	*p*
Intercept	0.668	0.232	24.11	2.88	0.008
Sex (Male)	−0.257	0.306	22.86	−0.84	0.41
Age_c	0.018	0.018	18.27	1.03	0.32
BARS_c	−0.058	0.034	20.76	−1.70	0.11
GDS_c	0.307	0.187	18.28	1.65	0.12
Age_c × BARS_c	0.011	0.004	18.38	3.08	0.006

Model-fit metrics were consistent across the two retained interaction models, indicating modest explanatory power of the fixed effects and substantial between-person heterogeneity captured by random intercepts. For Age × GDS, the marginal and conditional R^2^ were 0.114 and 0.376, respectively; for Age × BARS, they were 0.141 and 0.358. The ICC from a random-intercept-only model was 0.331, indicating that approximately one-third of the total variance resided between individuals.

## 4 Discussion

This study provides, to our knowledge, the first day-level mixed-effects evaluation of the cortisol-to-DHEAS awakening response ratio in PWD and their family caregivers, using paired awakening samples across multiple days. We found no between-group difference in cortisol-to-DHEAS awakening response ratio after accounting for within-person clustering and age, and this null contrast proved robust across an extensive series of sensitivity and specification checks. Within PWD, however, cortisol-to-DHEAS awakening response ratio showed positive effect modification by clinical burden: the association between age and the cortisol-to-DHEAS awakening response ratio strengthened at higher dementia severity (GDS) and at higher agitation (BARS), indicating positive Age × GDS and Age × BARS interactions. Together, these findings suggest that while caregivers and PWD do not differ on average in awakening HPA-axis balance, intra-PWD heterogeneity linked to symptom burden is salient and clinically meaningful.

Contrary to our priori hypothesis, mixed-effects analyses revealed no significant group difference between PWD and their family caregivers in the awakening hormonal stress ratio after adjusting for within-participant clustering, age, and sex. This null finding suggests that, despite distinct caregiving-related stress exposures, the diurnal HPA–adrenal balance indexed by ARC/ARDHEAS may remain broadly comparable between PWD and caregivers when chronic disease status and demographic factors are properly controlled. It is also possible that opposing (or compensatory) movements of cortisol and DHEAS render the ratio statistically neutral at the group level. Disaggregating components in future work (e.g., separate modeling of awakening cortisol and awakening DHEAS trajectories) may uncover patterned dysregulations that the ratio alone can mask.

Neuropsychiatric symptoms, such as agitation, apathy, depression, and anxiety, are among the most distressing features of dementia and may reflect underlying dysregulation of stress-response systems**.** Within the PWD subgroup, exploratory interaction models revealed that age moderated the associations of both dementia severity and agitation with the cortisol-to-DHEAS awakening response ratio. The exploratory interaction models revealed that age moderated the associations of both dementia severity and agitation with the cortisol-to-DHEAS awakening response ratio. Specifically, the positive Age × GDS and Age × BARS interactions indicate that older adults with more severe cognitive impairment or greater agitation exhibit a steeper positive age–ratio slope. This suggests that the combined burden of advanced age and greater neuropsychiatric impairment may exacerbate dysregulation of HPA–adrenal function, reflected in a higher cortisol-to-DHEAS balance upon awakening. They further imply that neuroendocrine imbalance in dementia is not uniform but depends on the intersection of age-related vulnerability and clinical symptom burden.

These findings highlight the value of modeling interactive rather than main effects, as they reveal subtle yet biologically meaningful sources of variability within PWD that may be masked in between-group comparisons. The age‐contingent associations broadly align with prior work showing that dementia is accompanied by HPA‐axis dysregulation and an age‐related shift toward higher cortisol/DHEA(S) ratios, particularly in individuals with greater neuropsychiatric burden [41]. Longitudinal cerebrospinal fluid (CSF) studies have likewise reported that higher cortisol levels are linked to more severe and worsening neuropsychiatric symptoms over time, independent of core Alzheimer's pathology [42]. Our findings contribute to this literature by demonstrating that, at the level of the cortisol-to-DHEAS awakening response ratio, age interacts with both dementia severity and agitation, highlighting interactive rather than uniform patterns of HPA-axis disruption. The findings suggest that neuroendocrine responses to behavioral symptoms may vary along a functional continuum, potentially reflecting stress thresholds or compensatory failure in later stages of the disease.

Recent reviews have emphasized that the cortisol awakening response reflects a distinct, awakening-entrained process regulated by multiple physiological and contextual factors rather than a unidirectional stress index [43]. Accordingly, we interpret the cortisol-to-DHEAS awakening response ratio as an index of relative coupling between morning glucocorticoid mobilization and DHEAS-linked counter-regulatory processes, recognizing that cortisol awakening response dysregulation can manifest as either elevated or blunted responses. A low cortisol awakening response may reflect chronic-stress-related blunting, which could plausibly occur among both PWD and caregivers; however, in our cohort, prior analysis of the diurnal rhythm (first-morning vs evening cortisol) showed a low prevalence of “flat curves,” suggesting preserved daily variation [28]. Within this context, the positive association between greater dementia severity and a higher cortisol-to-DHEAS awakening ratio in PWD is most parsimoniously read as reduced balance—i.e., relatively stronger morning glucocorticoid mobilization without a commensurate DHEAS response—while recognizing cortisol awakening response's directionality is context-dependent. Broader aging literature has documented relative declines in adrenal androgens and an altered cortisol/DHEA balance with advancing age, which has been linked to reduced stress adaptability and increased neurotoxic vulnerability [41].

CSF studies provide converging evidence for this pattern. A longitudinal investigation in memory clinic patients found that higher CSF cortisol levels were associated with more severe neuropsychiatric symptoms at baseline—particularly depression, anxiety, and apathy—and predicted worsening symptom burden over a three-year follow-up period, independent of AD pathology [42]. These findings suggest that cortisol may be a generalizable biomarker of neuropsychiatric vulnerability across biological compartments.

In addition, age-related changes in adrenal function—most notably the well-established decline in DHEAS production with aging [18,19,41] —may interact with dementia subtype. The findings in the present study on PWD may be complicated because different types of dementia are typical of certain ages. The hormonal regulatory patterns may be more disturbed in AD, which is more common among younger PWD, whereas those with cerebrovascular dementia in older PWD may have less general hormonal disturbance. Therefore, the observed interaction between age and agitation/dementia severity may partly reflect diagnosis-by-age interactions. This underscores the need for future studies to stratify dementia subtypes and integrate diagnostic heterogeneity into hormonal modeling [44].

Cortisol, essential for stress adaptation, can adversely affect aging when dysregulated, while DHEA(S) possesses properties that may mitigate these effects [3]. This study contributes to the literature by examining the balance between energy mobilization (cortisol) and regeneration (DHEAS) during the awakening response in older individuals with and without dementia. Going from sleep to wakefulness in the morning is a pronounced change. This is one of the underlying reasons why the rise of cortisol concentration in blood and saliva has been studied in relation to psychosocial stress. In the healthy individual, an increase in cortisol over the first 15–30 min after awakening is thought to reflect an anticipatory mobilization of energy in preparation for the upcoming day and may be larger when higher demands or stress are expected [45,46]. Importantly, the magnitude of the cortisol awakening response varies with anticipated demands for the coming day—typically larger on high-demand days [47] and smaller when a calm day is expected [48]. Conversely, in some conditions, a slight cortisol rise or even a downward slope during the first minutes after awakening could mirror the expectation of a calm day or it could also mirror an exhaustion of the regulatory adaptability. In more extreme cases, such dysregulation may coincide with flatter diurnal cortisol rhythms characterized by low morning and high evening levels of cortisol. In our cohort, however, prior analyses demonstrated that flat curves were not prevalent [28], indicating generally preserved circadian variation. Within this context, we therefore assume that in the present study, a considerable morning rise indicates the expectation of a more demanding or stressful experience, whereas persons with a slight increase expect a calm day.

DHEAS and cortisol are excreted from the adrenal cortex in acute stress situations. As pointed out above, DHEAS has a protective role. Under normal circumstances, a pronounced cortisol reaction should be “covered” by a correspondingly pronounced DHEAS reaction. However, there is a substantial decrease in DHEAS production with increasing age. A weakened DHEAS coverage during morning stress may worsen the adverse cortisol effects during stress in old age. The combination of a significant cortisol increases and, simultaneously, a small (or no) DHEAS increase in the morning during the first 15 min of wakefulness is not beneficial. Finally, the biological role of DHEAS remains incompletely understood. While often overlooked compared to cortisol, DHEAS may exert protective effects in brain aging and has shown therapeutic potential in age-related disorders [49]. Our findings reinforce the need to consider DHEAS not merely as a denominator in ratio calculations, but as an active hormonal agent worthy of independent investigation.

Methodologically, this study extends previous literature by incorporating multi-day, day-level awakening sampling and participant random intercepts to account for within-person clustering [50], providing a more conservative and statistically rigorous estimate of group differences. The robustness of this null group contrast across exclusion of single-day participants, leave-one-participant-out, trimming, extreme-residual removal, dropping influential clusters, alternative age parameterizations, and parametric bootstrap supports the stability of the result. These converging findings increase confidence that the absence of a group difference is not an artifact of particular participants, extreme observations, or model specification. At the same time, identifying significant age-by-symptom interactions within PWD demonstrates the added value of flexible mixed-effects modeling for revealing clinically contingent neuroendocrine variability beyond simple mean contrasts.

### 4.1 Clinical implications of findings

Given its non-invasive nature, wider accessibility, and cultural acceptability, particularly in low-resourced settings, saliva is a biofluid complementary to blood and CSF [51]. Studying DHEAS and cortisol in concert is supported by physiological data on their co-synthesis in the adrenal cortex. Still, they exert antagonistic effects: cortisol promotes catabolism and stress responsiveness, whereas DHEAS supports neuroprotection, regeneration, and anabolic balance [11]. In prior work, higher morning cortisol-to-DHEAS ratios have been proposed as sensitive indicators of HPA-axis function and endocrine aging, reflecting the balance between catabolic and anabolic processes [3]. Building on this literature, the present study focuses on the cortisol-to-DHEAS awakening response ratio as a dynamic, awakening-entrained index of neuroendocrine balance. Importantly, this awakening response ratio is conceptually distinct from single-sample cortisol/DHEAS ratios, as it captures the relative coupling of changes in cortisol and DHEAS from awakening to 15 min later rather than absolute hormone concentrations at one time point. Evidence that single-sample cortisol/DHEAS ratios are sensitive markers of HPA-axis activity, therefore motivated—but does not predetermine—our examination of the cortisol-to-DHEAS awakening response ratio. Our data indicate that this awakening response ratio is sensitive to clinical burden within PWD—showing higher values among older individuals with greater agitation and/or more advanced dementia severity—even when mean levels do not differ between PWD and caregivers.

Clinically, this points to the utility of the cortisol-to-DHEAS awakening response ratio as a candidate stratified monitoring biomarker in dementia care. Repeated assessment of the ratio could help delineate subgroups of PWD at heightened risk for stress-related dysregulation—for example, older individuals with higher agitation levels or more advanced dementia severity—and could be used to track response to interventions that target behavioral symptoms or enhance stress-buffering capacity (including approaches that may bolster DHEA(S) pathways and other non-pharmacological approaches). At the same time, the present study is exploratory and underpowered for small effects, so the monitoring value of the cortisol-to-DHEAS awakening response ratio should be viewed as hypothesis-generating and requires replication in larger, well-characterized cohorts.

### 4.2 Limitations

First, saliva samples were collected based on participants' natural diurnal rhythms rather than fixed clock times, and the exact moment of awakening was not objectively verified using wearable devices or electroencephalography (EEG) [52]. Although this approach reflects common practice in field-based biomarker studies, it introduces variability in estimating the true awakening response. Notably, while our protocol did not adhere to the standardized procedures for awakening response assessment, this deviation likely has less impact on calculating the cortisol-to-DHEAS ratio than on the absolute quantification of each hormone's response individually. Future studies should verify awakening using wearable devices (e.g., actigraphy) or EEG, employ time-stamped collection devices and narrow allowable sampling windows, and include actual sampling times as quality-control flags. Additionally, our 0–15-min window does not capture the later 30–45-min peak [45], so part of the awakening response was not observed. Because the primary outcome is a ratio (awakening cortisol/awakening DHEAS), truncation may partly cancel if it scales both components similarly; nevertheless, given potentially non-parallel cortisol and DHEAS kinetics, any residual impact would likely attenuate group and symptom differences. And it is not known whether the rise during the first 15 min is slower or faster than during the subsequent 15 min in our specific population, and the DHEAS kinetics over this window are even less certain. Future work should include +30/+45-min sampling with objective awakening verification to characterize both the early mobilization and the later peak.

Second, we did not systematically capture medication class, dose, timing, or treated-versus-untreated status; consequently, we were unable to adjust for medication use. Several medication classes that are common in dementia care may influence HPA-axis dynamics or autonomic responsivity, including antidepressants, beta-blocking agents, thyroid hormone replacement therapies, antipsychotics, anti-inflammatory medications, and systemic or inhaled glucocorticoids [50,[53], [54], [55], [56], [57], [58]]. In our previous work in a related cohort of PWD and their family caregivers, we observed that participants often used multiple medications simultaneously—including AD medications alongside beta-blockers, antidepressants, and thyroid hormone replacement—and that such complex regimens were typical in this population [50]. These patterns of multimorbidity and polypharmacy may contribute to individual differences in hormonal responsivity, and residual confounding by unmeasured medication effects cannot be excluded. Future studies should document medication exposures with sufficient granularity (e.g., class, dose, and timing relative to sampling) to enable covariate adjustment or sensitivity analyses.

Third, the number of level-2 units (*n* = 28 PWD IDs), which constrains power for detecting small effects—particularly cross-level interactions—and precluded inclusion of random slopes or highly flexible smooths. We mitigated this by using random-intercept models with small-sample df corrections, restricting model complexity, screening interactions one-at-a-time, and stress-testing results via an extensive sensitivity program and a parametric bootstrap. Notably, the null PWD–caregiver contrast was stable across all checks (GMRs ≈1 with CIs spanning unity), suggesting the lack of a group difference is not an artifact of small-sample idiosyncrasies. By contrast, the Age × GDS and Age × BARS interactions, while statistically significant after safeguards, should be interpreted as hypothesis-generating and warrant replication in larger PWD samples.

Fourth, we did not administer an anxiety measure. Given known links between anxiety and HPA-axis activity, residual confounding by unmeasured anxiety cannot be ruled out. Future studies could include a brief, validated anxiety assessment.

Fifth, collection device differed by role (swab for PWD, passive drool for caregivers). While studies support cross-device comparability and prediction of serum indices, device heterogeneity could add noise and likely attenuate PWD-vs-caregiver contrasts [35]; future work should harmonize collection methods within roles and/or implement device-adjusted sensitivity analyses.

Finally, this study focused on HPA-axis hormones measurable in saliva at awakening (cortisol and DHEAS); catecholamines (e.g., epinephrine, norepinephrine), which primarily index sympathetic-adrenal-medullary (SAM) activity and are difficult to quantify reliably in home saliva sampling [59,60], were not included—an omission that should be considered when interpreting the findings.

## 5 Conclusions

Using multi-day, day-level sampling and linear mixed-effects models that accounted for within-participant clustering and age, we found no average difference in the cortisol-to-DHEAS awakening response ratio between PWD and family caregivers. In PWD, however, the ratio displayed heterogeneity: the association with age strengthened at higher dementia severity and greater agitation, indicating that neuroendocrine balance upon awakening varies with PWD and links to symptom burden. These findings suggest that the cortisol-to-DHEAS awakening response ratio is most informative for characterizing within-PWD variability, pointing to age-by-symptom contingencies as a plausible target for phenotyping stress-related vulnerability in dementia. Replication in larger cohorts—with objective awakening verification, harmonized sampling devices, and covariates for medication and anxiety—is warranted. Evaluating cortisol and DHEA(S) as separate signals in parallel with their ratio, and selecting biological matrices (such as hair, sweat, plasma, and urine) aligned to temporal scope (e.g., saliva for day-to-day dynamics; hair for longer-term variations), may refine biological interpretation and clarify when the ratio flags meaningful HPA–adrenal dysregulation relevant to monitoring and individualized intervention.

## CRediT authorship contribution statement

**Wanrui Wei:** Writing – review & editing, Writing – original draft, Visualization, Validation, Software, Methodology, Formal analysis, Conceptualization. **Töres Theorell:** Writing – review & editing, Writing – original draft, Validation, Methodology, Conceptualization. **Gabriella Engstrom:** Writing – review & editing, Project administration, Investigation, Data curation, Conceptualization. **Azita Emami:** Writing – review & editing, Supervision, Resources, Project administration, Methodology, Investigation, Funding acquisition, Conceptualization.

## Ethical approval

Study procedures were conducted in accordance with the Declaration of Helsinki under the approval of the Karolinska Institute Institutional Review Board (Dnr: 2018/1596-31/2). All participants completed the informed consent process prior to initiation of any study procedures. Informed consent was obtained from all participants in the study. The study was performed in line with the principles of the Helsinki Declaration.

## Funding

The author(s) disclosed receipt of the following financial support for the research, authorship, and/or publication of this article: This work was supported by AMF Insurance Company in Sweden, Section of Elderly Research (Emami) and the Robert G. and Jean A. Reid endowed fund (Emami) from the University of Washington School of Nursing.

## Declaration of competing interest

The authors declare that they have no known competing financial interests or personal relationships that could have appeared to influence the work reported in this paper.
