# Supplementary material for: Cortisol-to-DHEAS awakening response ratio in people with dementia and family caregivers: Associations with age, dementia severity and agitation
Source: Compr Psychoneuroendocrinol. 2025 Dec 19;25:100334. doi: 10.1016/j.cpnec.2025.100334 (PMC12818106; doi:10.1016/j.cpnec.2025.100334)
Supplement: Multimedia component 1 [file mmc1.docx]

Table of Contents

[Supplementary Table 1. Sensitivity and specification checks for the main group contrast. 2](#_Toc212508173)

[Supplementary Table 2. Kenward–Roger Type-III tests for the corresponding interaction models (Base + Age×GDS). 3](#_Toc212508174)

[Supplementary Table 3. Kenward–Roger Type-III tests for the corresponding interaction models (Base + Age×BARS). 4](#_Toc212508175)

[Supplementary Figure 1. Predicted geometric mean of ARC/ARDHEAS by Group from the linear mixed-effects model with natural splines for age (df=4). 5](#_Toc212508176)

| Supplementary Table 1. Sensitivity and specification checks for the main group contrast. | | | |
| --- | --- | --- | --- |
| Scenario | GMR | 95% CI | *p* value |
| Main (ns(Age,df=4)) | 0.97 | [0.64, 1.46] | 0.87 |
| S1: drop single-day IDs | 1.02 | [0.68, 1.53] | 0.93 |
| S2: LOCO | 0.97 | [0.92, 1.03] | 0.667–0.985 |
| S3: trim 1% tails | 0.92 | [0.63, 1.36] | 0.69 |
| S4: remove | Pearson>3 |  | 0.95 |
| S5: drop 2 most influential IDs | 0.87 | [0.58, 1.31] | 0.50 |
| Spec: linear Age | 0.96 | [0.63, 1.46] | 0.84 |
| Spec: ns(Age,df=3) | 0.97 | [0.64, 1.45] | 0.87 |
| Spec: ns(Age,df=5) | 0.92 | [0.62, 1.37] | 0.68 |
| Bootstrap | 0.98 | [0.82, 1.16] | — |

*Note.*

- S1: drop single-day IDs

- S2: leave-one-participant-out (LOCO)

- S3: trim 1% tails

- S4: remove |Pearson|>3

- S5: drop 2 most influential IDs

- Spec: linear Age

- Spec: ns(Age,3)

- Spec: ns(Age,5)

- Bootstrap (199 runs)

| Supplementary Table 2. Kenward–Roger Type-III tests for the corresponding interaction models (Base + Age×GDS). | | |
| --- | --- | --- |
| Effect | F | *p* |
| Sex | 0.003 | 0.96 |
| Age_c | 0.685 | 0.42 |
| GDS_c | 0.493 | 0.49 |
| BARS_c | 1.156 | 0.30 |
| Age_c×GDS_c | 4.970 | 0.04 |

| Supplementary Table 3. Kenward–Roger Type-III tests for the corresponding interaction models (Base + Age×BARS). | | |
| --- | --- | --- |
| Effect | F | *p* |
| Sex | 0.702 | 0.41 |
| Age_c | 1.054 | 0.32 |
| BARS_c | 2.848 | 0.11 |
| GDS_c | 2.691 | 0.12 |
| Age_c×BARS_c | 9.456 | 0.007 |


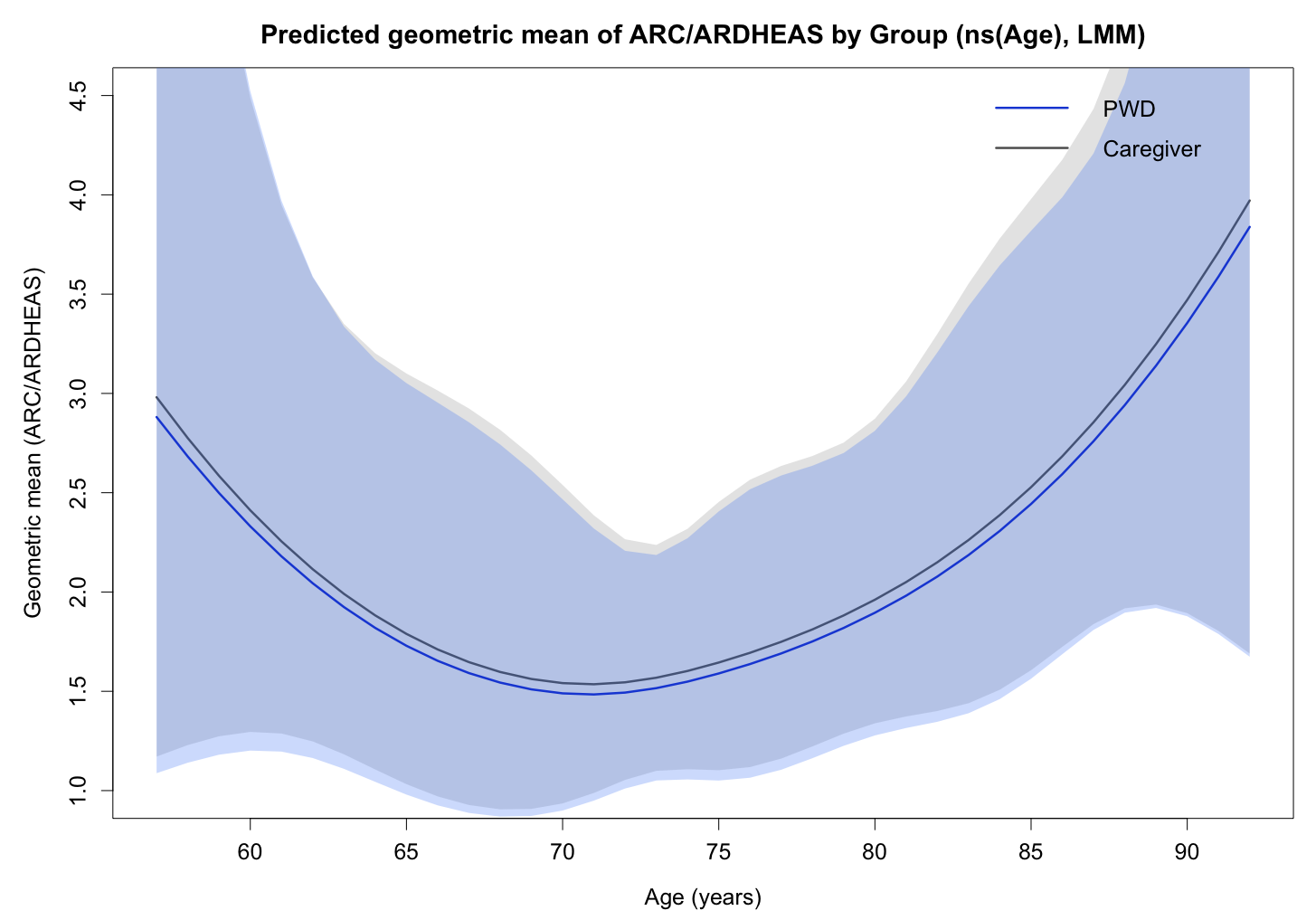


Supplementary Figure 1. Predicted geometric mean of ARC/ARDHEAS by Group from the linear mixed-effects model with natural splines for age (df=4).

*Note: Plotted values age- and sex-adjusted marginal means estimated from the linear mixed-effects model (Group + Sex + ns(Age, df = 4) + random intercept for ID), back-transformed from the log scale. Shaded bands show 95% confidence intervals. The curves for PWD and caregivers substantially overlap across the age range, indicating no systematic group difference after accounting for within-participant clustering and non-linear age effects.*
